# Study on the mechanism of American ginseng extract for treating type 2 diabetes mellitus based on metabolomics

**DOI:** 10.3389/fphar.2022.960050

**Published:** 2022-09-02

**Authors:** Tiantian Liu, Dan Wang, Xinfeng Zhou, Jiayin Song, Zijun Yang, Chang Shi, Rongshan Li, Yanwen Zhang, Jun Zhang, Jiuxing Yan, Xuehui Zhu, Ying Li, Min Gong, Chongzhi Wang, Chunsu Yuan, Yan Cui, Xiaohui Wu

**Affiliations:** ^1^ School of Pharmacy, Tianjin Medical University, Tianjin, China; ^2^ Department of Pharmacy, Chu Hisen-I Memorial Hospital, Tianjin Medical University, Tianjin, China; ^3^ Tianjin Neurological Institute, Tianjin Medical University, Tianjin, China; ^4^ Tang Center for Herbal Medicine Research, University of Chicago, Chicago, IL, United States; ^5^ Department of Pathogen Biology, School of Basic Medical Sciences, Tianjin Medical University, Tianjin, China

**Keywords:** American ginseng extract, metabolomics, type 2 diabetes mellitus, potential biomarkers, metabolic pathways

## Abstract

American ginseng extract (AGE) is an efficient and low-toxic adjuvant for type 2 diabetes mellitus (T2DM). However, the metabolic mechanisms of AGE against T2DM remain unknown. In this study, a rat model of T2DM was created and administered for 28 days. Their biological (body weight and serum biochemical indicators) and pathological (pancreatic sections stained with HE) information were collected for further pharmacodynamic evaluation. Moreover, an ultra-performance liquid chromatography–mass spectrometry–based (UHPLC–MS/MS–based) untargeted metabolomics method was used to identify potential biomarkers of serum samples from all rats and related metabolic pathways. The results indicated that body weight, fasting blood glucose (FBG), fasting blood insulin (FINS), blood triglyceride concentration (TG), high-density lipoprotein cholesterol (HDL-C), insulin resistance index (HOMA-IR) and insulin sensitivity index (ISI), and impaired islet cells were significantly improved after the high dose of AGE (H_AGE) and metformin treatment. Metabolomics analysis identified 101 potential biomarkers among which 94 metabolites had an obvious callback. These potential biomarkers were mainly enriched in nine metabolic pathways linked to amino acid metabolism and lipid metabolism. Tryptophan metabolism and glutathione metabolism, as differential metabolic pathways between AGE and metformin for treating T2DM, were further explored. Further analysis of the aforementioned results suggested that the anti-T2DM effect of AGE was closely associated with inflammation, oxidative stress, endothelial dysfunction, dyslipidemia, immune response, insulin resistance, insulin secretion, and T2DM-related complications. This study can provide powerful support for the systematic exploration of the mechanism of AGE against T2DM and a basis for the clinical diagnosis of T2DM.

## Introduction

The prevalence of diabetes, a chronic disease that is both serious and common in this day and age, is increasing every year. According to statistics, the global prevalence of diabetes in people aged 20–79 years is estimated at 10.5% in 2021 and will rise to 12.2% by 2045 (Ogurtsova et al., 2022). , It should be noted that type 2 diabetes accounts for more than 90% of patients with diabetes (Chatterjee et al., 2017). It is well known that the typical feature of T2DM is hyperglycemia mainly induced by insulin resistance (Borse et al., 2021). A variety of serious symptoms such as blindness, kidney failure, heart attacks, and so on caused by T2DM can greatly shorten patients’ life (Nasykhova et al., 2020). Most western drugs can bring about great side effects while performing effective anti-T2DM effects (Lee et al., 2021). Surprisingly, many plant extracts have a better anti-T2DM effect and lower side reaction than modern medicines (Tran et al., 2020). Given this, traditional Chinese medicine extracts have wide application prospects in the clinical treatment of T2DM and are promising to develop new drugs.

American ginseng is one of the well-known ginseng species originating from North America, which has multiple medicinal active ingredients such as ginsenosides, polyacetylenes, polyphenolic compounds, and so on (Punja, 2011). Ginsenosides, as its major active component, play a crucial role in treating cancer, type 2 diabetes, cardiovascular diseases, and central nervous system diseases (Qi et al., 2011). Research studies have proven that AGE has an antioxidant effect, reduce glucose levels, and improve insulin sensitization (Yin et al., 2008). AGE can promote insulin production and prevent β-cell loss by inhibiting mitochondrial uncoupling protein-2 (UCP-2), increasing the ATP level and anti-apoptotic factor B-cell lymphoma-2 (Bcl-2), and downregulating pro-apoptotic factor caspase-9 (Luo and Luo, 2006). Furthermore, AGE was acknowledged as an efficient and low-toxic adjunct for T2DM (Vuksan et al., 2019). Consequently, it is of great significance to study the mechanism of AGE against T2DM based on the metabolic level.

Metabonomics is a popular discipline dedicated to detecting all small-molecule metabolites stemming from biological systems based on all kinds of analytical methods (Liggi and Griffin, 2017). Its application in mass spectrometry (MS) analysis is more extensive than genomics and proteomics providing the benefit of higher precision, a lower sample number, etc. In addition, because of its high sensitivity and wide detection range, liquid chromatography–mass spectrometry (LC–MS) was deemed to be a more powerful method to screen potential markers from biological samples (Ma et al., 2018). In addition, metabonomics technology can facilitate the process of discovering and developing novel drugs through the identification of molecular biomarkers.

In our research, a UHPLC–MS/MS-based untargeted metabolomics method was applied to study the mechanism of AGE against T2DM by identifying its potential biomarkers and metabolic pathways.

## Materials and methods

### Chemicals and reagents

The standards of HPLC grade ginsenoside Rb1, Re, Rg1, Rc, and Rd (Shanghai Yuanye Bio-Technology Co., Ltd.). HPLC grade acetonitrile and methanol (Tianjin Kemio Chemical Reagent Co., LTD.). Ultrapure water (A Milli-Q water purification system; Merck, America). Blood glucose test strips (Roche Diabetes Care GmbH). Glucose assay kit, triglyceride assay kit, and high-density lipoprotein cholesterol assay kit (Nanjing Jiancheng Bioengineering Institute). Rat INS (Insulin) ELISA kit (Elabscience Biotechnology Co., Ltd.).

### Preparation of the AGE and quality control

American ginseng (Beijing Tong-Ren-Tang Pharmaceutical Co., Ltd., No. 20190601) was crushed, weighed, and extracted with 70% ethanol by Soxhlet reflux. With the solvent of extract evaporated under a vacuum at 50°C, the concentrates were collected. Applying a freeze dryer lyophilized these concentrates to acquire AGE in a powder form. The yield of the extract was ∼ 39%. Furthermore, the content of ginsenosides from AGE was measured using an Agilent 1200 HPLC System (Bruker, America) and an anti-phase C18 column (Welch, Shanghai). The mobile phase comprised [A] water and [B] acetonitrile. The followings are the gradient elution conditions: 0 min, 17.5%; 0–20 min, 21%; 20–40 min, 26%; 40–42 min, 40%; and 42–50 min, 50%. The flow rate was set to 1.0 ml/min, and the detection wavelength was set at 203 nm.

### Induction of experimental diabetes mellitus in mice

Male SD rats (180∼220 g) were offered by Beijing Vital River Co., Ltd. After adaptive feeding for 1 week, the rats were separated into the control group and model group at random. The two groups were, respectively, treated with a common feed and a high-fat feed (comprising 20% sucrose, 10% lard, 5% yolk powder, 2.5% cholesterol, 1% sodium cholate, and 66.5% maintenance feed, purchased from Beijing XiaoshuYoutai Biotechnology Co., Ltd.). After being treated with high-fat feed for 8 weeks, the model rats were injected intraperitoneally with 33 mg/kg of streptozotocin (STZ) (Gentihold, Beijing). The control rats were injected with 1 ml/kg citrate buffer (Suolaibao, Beijing). By taking blood samples from the tail vein, we tested FBG values by applying blood glucose test strips every week. Ultimately, those rats with FBG >16.7 mmol/L were separated into four groups at random, including the model (sodium chloride injection: 5 ml/kg/d) group, metformin (0.2 g/kg/d) group, low-dose American ginseng extract (L_AGE) (1.5 g/kg/d) group, and high-dose American ginseng extract (H_AGE) (3.0 g/kg/d) group. The aforementioned experiment was in accordance with the Animal Ethics Committee of the Institute of Radiation Medicine, Chinese Academy of Medical Sciences and the approval number is IRM-DWLL-2020148.

### Histopathological observation and biochemical index detection

The rats were fasted overnight (12 h) after 4 weeks of administration and anesthetized with 10% chloral hydrate. Blood was obtained from the abdominal aorta using a 5 ml vacuum blood collection tube (containing inert separator gel and procoagulant). Then the samples were left to stand for 30 min and centrifuged at 3,000 rpm for 15 min. The supernatant was dispensed into 2 ml lyophilized tubes, snap-frozen in liquid nitrogen for 10 min and placed in a foam box with dry ice, and finally transferred to a −80°C refrigerator for storage. The pancreas of rats was isolated, fixed with 4% paraformaldehyde, dehydrated, translucent, embedded in paraffin, sectioned, and stained. The staining material was hematoxylin–eosin. Pancreatic pathology was observed using an optical microscope (Leica DMI3000B, Germany) with a magnification of ×400. The efficacy of AGE was evaluated by comparing the area and shape of islets between groups.

In addition, the serum samples of every group were also used to test fasting blood glucose (FBG), fasting blood insulin (FINS), blood triglyceride concentration (TG), and high-density lipoprotein cholesterol (HDL-C). The insulin resistance index (HOMA-IR) and insulin sensitivity index (ISI) were also reckoned to assess the efficacy of AGE. HOMA-IR = (FBG, mmol/L) × (FINS, mIU/L)/22.5; ISI = 1/(FBG, mmol/L) × (FINS, mIU/L).

### Sample pretreatment

A measure of 100 µL of the serum sample was accurately weighed into a 1.5-ml centrifuge tube, 400 µL of methanol:acetonitrile (1:1, v/v) solution containing 0.02 mg/ml of internal standard (L-2-chlorophenylalanine) was added, then vortexed and mixed for 30 s, followed immediately by low-temperature sonication for 30 min (5°C, 40 KHz). The samples were left at −20°C temperature for 30 min and centrifuged for 15 min (13000 g, 4°C). The supernatant was removed and blown dry with nitrogen. The residue was reconstituted with 100 μL of the reconstituted solution (acetonitrile: water = 1:1), then vortexed and mixed for 30 s, followed by low-temperature sonication for 30 min (5°C, 40 KHz) and centrifugation for 10 min (13,000 g, 4°C), and the supernatant was pipetted into the injection vial with an internal cannula for analysis. In addition, 20 µL of the supernatant for each sample was mixed as a quality control sample.

### UHPLC–MS/MS conditions

Using a Thermo UHPLC system (ACQUITY UPLC HSS T3: 100 mm × 2.1 mm i. d. 1.8 µm; Waters, Milford, United States) for completed chromatographic separation of the metabolites. Gradient elution comprised 0.1% formic acid in water:acetonitrile (95:5,v/v) [A] and 0.1% formic acid in acetonitrile:isopropanol:water (47.5:47.5:5,v/v) [B] at a flow of 0.4 ml/min as follows: 0–3.5 min, 24.5% B; 3.5–5, min 65% B; 5–5.5 min, 100% B; 5.5–7.4 min, 100% B; 7.4–7.6 min, 51.5% B; and 7.6–10 min, 0% B. The sample size was 2 μL, and the column was kept under 40°C temperature. A Thermo UHPLC-Q Exactive HF-X Mass Spectrometer provided with an electrospray ionization (ESI) source was used to obtain the mass spectrometric data. The heater and capillary temperatures were, respectively, set to 425 and 325°C. The sheath gas and aux gas flow rates were, respectively, set to 50 arb and 13 arb. The ion-spray voltage floating (ISVF) in positive and negative patterns was, respectively, set to −3500 and 3500 V. Full MS and MS/MS resolutions were, respectively, set to 60,000 and 7,500 using the data-dependent acquisition (DDA) mode acquisition data. The mass range was set to 70–1,050 m/z during the detection.

### Multivariate data processing and analysis

The original data obtained by UHPLC–MS detections were input into the Progenesis QI 2.3 (Nonlinear Dynamics, Waters, United States) for baseline filtering, peak identification, integration, retention time correction, peak alignment, normalization, and integration. The internal standard was used for data QC (reproducibility), while variables with relative standard deviations (RSDs) > 30% of the QC samples were excluded and log transformed data to obtain the final data matrix for subsequent analysis. The MS and MS/MS mass spectrometry information was matched with KEGG (https://www.kegg.jp/kegg/pathway.html), Human Metabolome Database (HMDB) (http://www.hmdb.ca/), and Metlin (https://metlin.scripps.edu/) databases to obtain metabolite information.

The pre-processed data were uploaded on the Majorbio Cloud Platform (https://cloud.majorbio.com) for data analysis. Principal component analysis (PCA), partial squares discriminant analysis (PLS-DA), and orthogonal least squares discriminant analysis (OPLS-DA) were performed using the ropls R package (Version1.6.2, http://bioconductor.org/packages/release/bioc/html/ropls.html). Also, 7-fold cross-validation and 200 permutation tests were used to assess the stability of the OPLS-DA model and to check whether it was overfitted. In addition, Student’s t-test analysis was performed. The selection of significantly different metabolites was determined based on the variable importance in the projection (VIP) obtained from the OPLS-DA model and the *p*-value of the Student’s t-test, with metabolites with VIP>1 and *p* < 0.05 being significantly different metabolites. The potential biomarkers were further screened based on KEGG and HMDB databases. Finally, the scipy.stats module in the Python software package was used for pathway enrichment analysis, and Fisher’s exact method was used to obtain the biological pathways most relevant to the experimental processing.

### Statistical analysis

The data obtained from the experiment are shown as the mean ± standard deviation (mean ± SD). We applied the GraphPad Prism 8.0.2 statistical analysis software to carry out Student’s t-test and two-way ANOVA. All *p*-values were two-tailed, and differences with *p*-values < 0.05 were considered statistically significant.

## Results

### Ginsenosides from AGE

The HPLC technology was used to determine the content of main ginsenosides from AGE. The following, respectively, represented the content of these ginsenosides (%): Rb1 (8.69), Re (3.34), Rd (1.36), Rc (0.96), and Rg1 (0.58) (Supplementary Figure S1).

### Weight observation results

**FIGURE 1:**
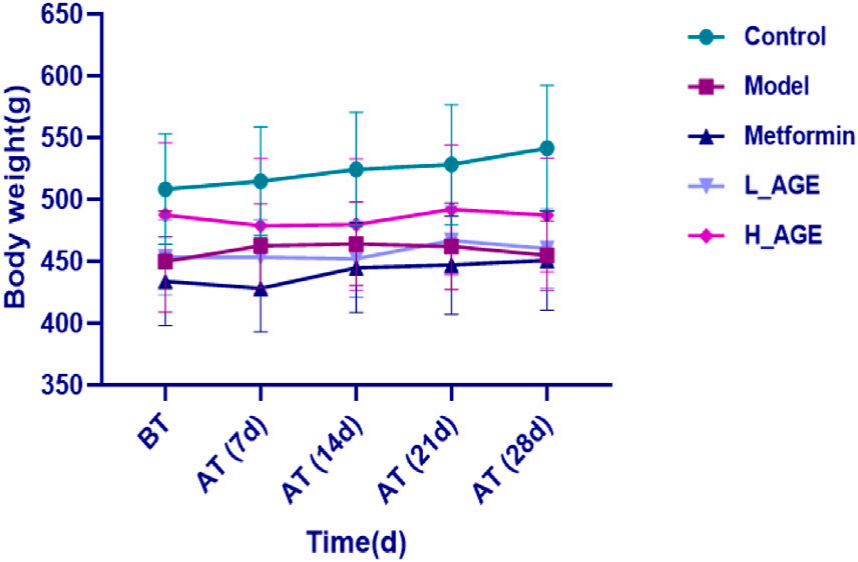
Body weight change of control, model, and treatment rats. Note: data are expressed as means ± SD (*n* = 6); BT: before treatment; AT: after treatment.

Figure 1 depicts the weight change trend of each group during administration. The rats in the control group and metformin had a gradual increase in weight. The weight of model rats first increased and then slowly decreased. Compared with the model group, the weight change in the L_AGE and H_AGE groups showed a clear opposite trend.

### Biochemical detection results

**FIGURE 2:**
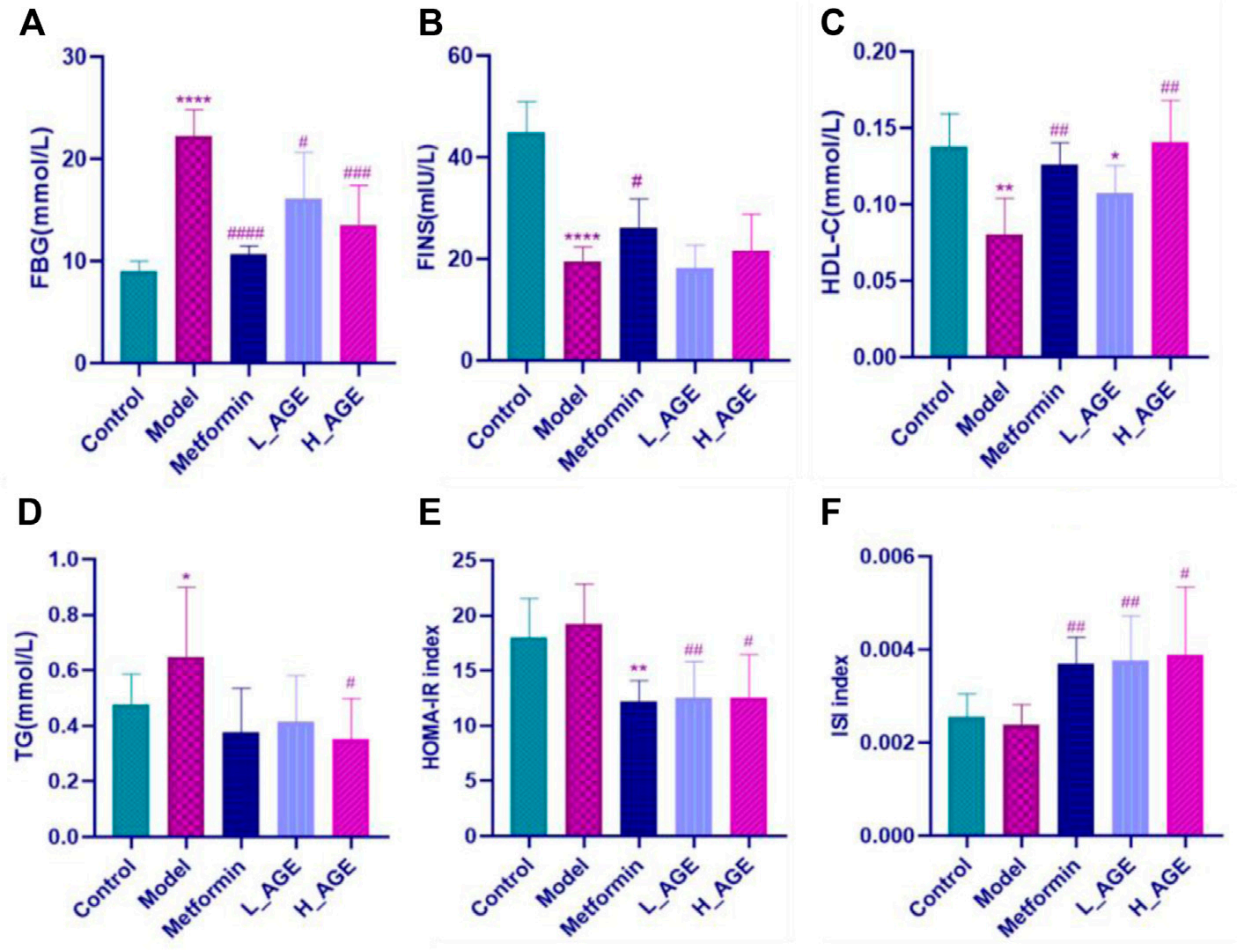
Serum biochemical marker levels in each group. **(A–F)** respectively represent the levels of fasting blood glucose (FBG), fasting blood insulin (FINS), high-density lipoprotein cholesterol (HDL-C), blood triglyceride concentration (TG), insulin resistance index (HOMA-IR) and insulin sensitivity index (ISI) in serum samples. Note: data are expressed as mean ± SD (*n* = 6); Student’s t-test: compared with the control group, ****p < 0.0001,***p < 0.001, **p < 0.01, and*p < 0.05; compared with the model group, ^####^ *p* < 0.0001,^###^ *p* < 0.001,^##^ *p* < 0.01, and ^#^ *p* < 0.05.

The levels of FBG, FINS, TG, and HDL-C in serum samples were evaluated or calculated to reflect the efficacy of administration. Figures 2A–F show that AGE and metformin improved the aforementioned biochemical markers compared with the model rats. Compared with rats in the AGE group, the FBG, FINS, and HOMA-IR index in metformin rats were better regulated; adversely, the HDL-C, TG, and ISI index were better modulated in AGE rats compared with the metformin rats.

### Pancreas section results

**FIGURE 3:**
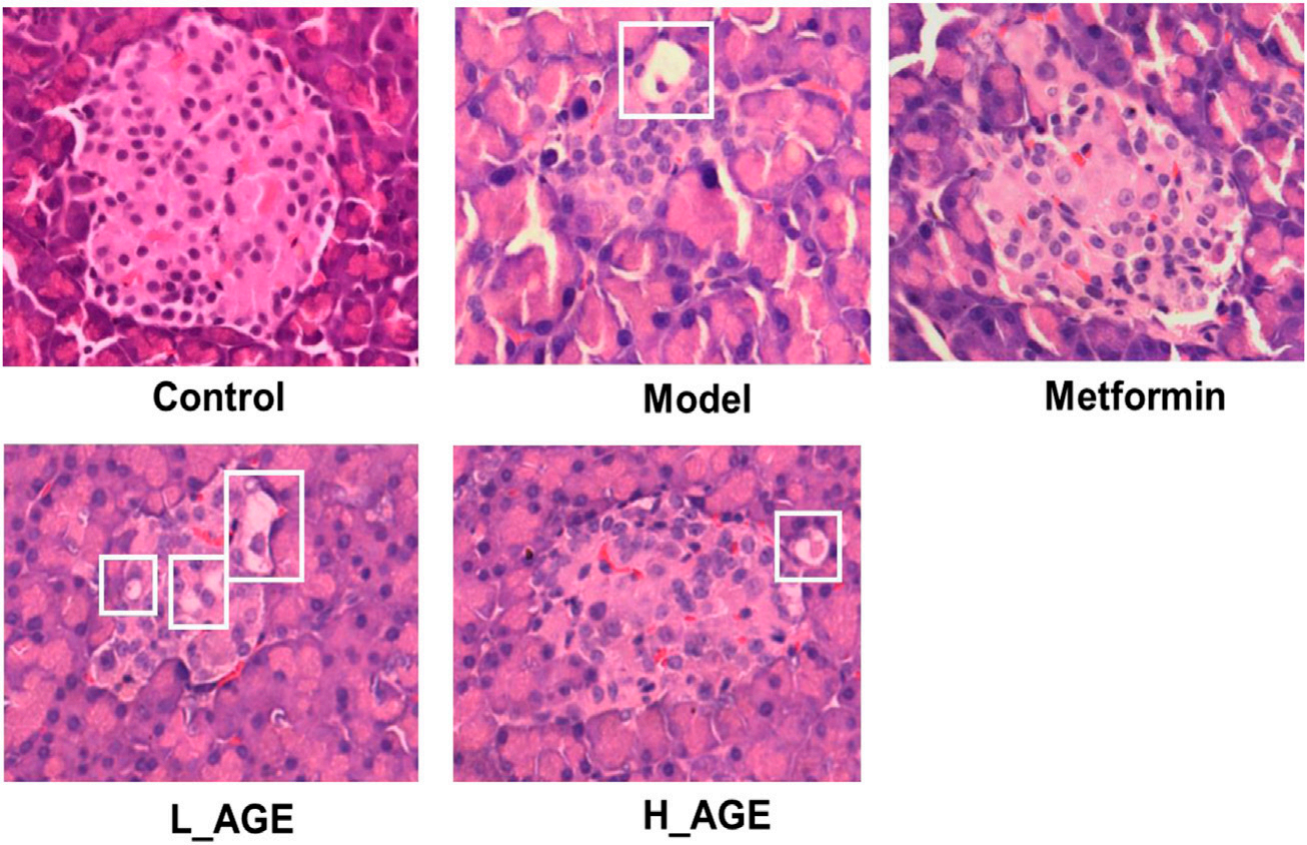
Pathological changes of H & E staining in pancreatic tissue of rats (×400).

To verify the protective effect of AGE, we observed the morphology and size of pancreatic islets in pancreatic sections (Figure 3). The islets of the model rats had fewer cells, smaller size, more irregular morphology, and vacuolar degeneration compared with those of the control rats. The islets of the rats in the L_AGE group were not significantly different from those of the model rats. The islets in the metformin and H_AGE groups were more regular in morphology, had a higher number of islet cells, did not have vacuolar degeneration, and tended to be at the level of the control group with a recovery percentage of about 40%–50% compared to the model rats.

### Metabolomic study of serum samples

#### The stability for UHPLC–MS/MS analysis

The total ion chromatograms (TIC) of quality control (QC) samples were obtained in the positive and negative ion modes. Taking sample QC01 as an example, the TIC (Supplementary Figure S2) consistently showed a good peak shape and relatively uniform distribution. It was verified that the LC–MS system was stable in the process of the whole detection.

#### Multivariate statistical analysis

**FIGURE 4:**
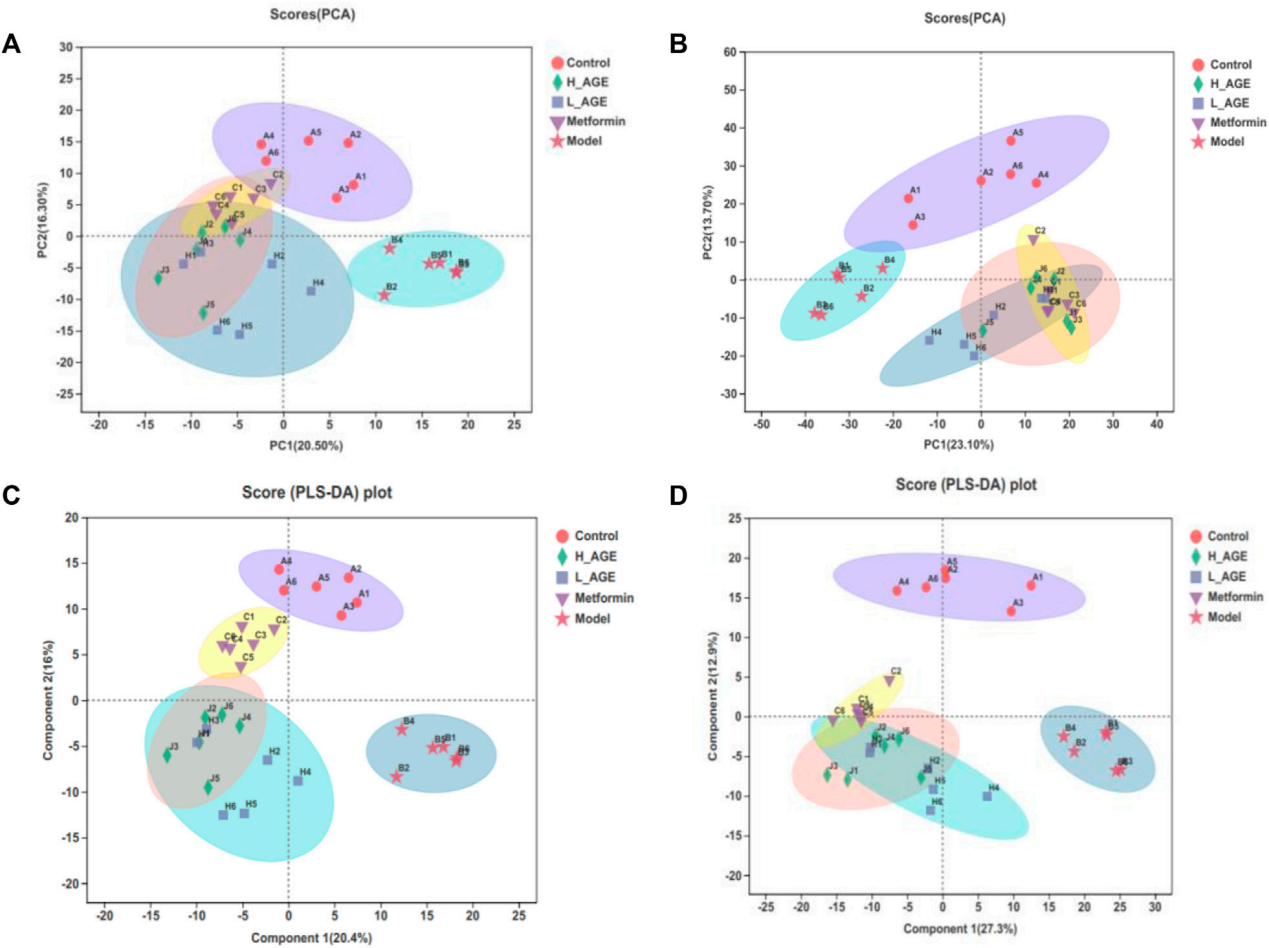
PCA score diagrams of the samples from control, model, and treatment rats in ESI + mode **(A)** or ESI- mode **(B)**. PLS-DA score diagrams of the samples from control, model, and treatment rats in ESI+ **(C)** or ESI- **(D)** modes. The ellipse expresses that the confidence interval is 95%.

To better unveil the distinction among samples of different groups, we simultaneously adopted the PCA and PLS-DA approaches. The closer the distance of dots in the diagram, the more similar the metabolite expression pattern of samples. As shown in Figures 4A–D, there was an obvious separation between the samples in the model rats and control rats; the metabolite samples in the treatment groups and model group also had an obvious deviation; the samples in metformin rats were more comparable to the control rats than those to the H_AGE rats, suggesting that the efficacy of metformin was more significant than that of H_AGE; the samples in H_AGE rats were more comparable to the control rats than those to the L_AGE rats, which indicated the more significant efficacy of H_AGE against T2DM than L_AGE.

**FIGURE 5:**
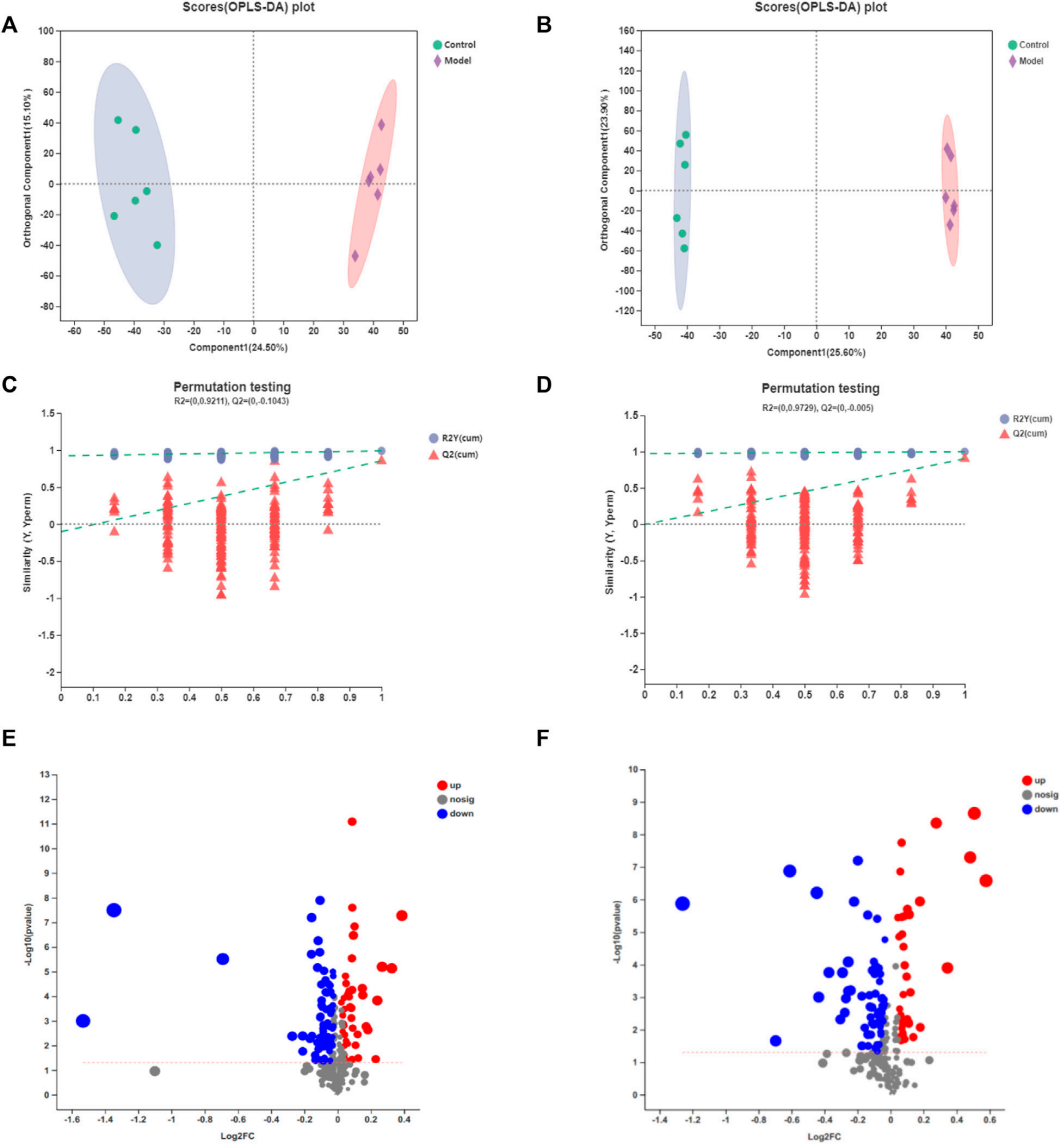
OPLS-DA score diagrams of the samples from control vs. model in the positive mode **(A)** or negative mode **(B)**; 7-fold cross-validation plot of the OPLS-DA model with 200 permutation tests in the positive mode **(C)** or negative mode **(D)**; volcano plots of the samples from control vs. model in the positive mode **(E)** or negative mode **(F)**. Ellipse expresses that the confidence interval is 95%.

In contrast with PLS-DA, OPLS-DA can greatly distinguish the differences between the two groups and improve the effectiveness and analytical capability of the model. Therefore, we adopted the OPLS-DA method to analyze the metabolomics data of samples on control vs. model and model vs. H_AGE group. Figures 5A–D and Supplementary Figures S3A–D display the results. The score diagrams for OPLS-DA presented a clear separation between the aforementioned two pairs of groups. Using the 7-fold cross-validation method gained R2X (cum), R2Y (cum), and Q2 (cum) of the OPLS-DA model to verify its reliability. The more these parameters are inclined to 1, the more the model is reliable. Q2 > 0.5 indicated the good predictive ability of the OPLS-DA model. For OPLS-DA models obtained from control and model groups, the three parameters, respectively, are 0.435, 0.994, and 0.91 in the positive ion mode and 0.509, 0.994, and 0.924 in the negative ion mode. Moreover, we used 200 response permutation testing (RPT) to estimate the accuracy of the OPLS-DA model, in which the left R2 and Q2 values were less than the right initial values, suggesting that the model was available.

The volcano plots (as shown in Figures 5E,F) and (Supplementary Figures S3E,F) showed the changing trend of the differential metabolite expression in model vs. control and H_AGE vs. model groups. Each dot represents a specific metabolite and the size of the dot indicates the VIP value. The larger the dot, the larger the VIP value. The red dot refers to the downregulated metabolites and the blue dot refers to upregulated metabolites.

#### Potential biomarker identification

**FIGURE 6:**
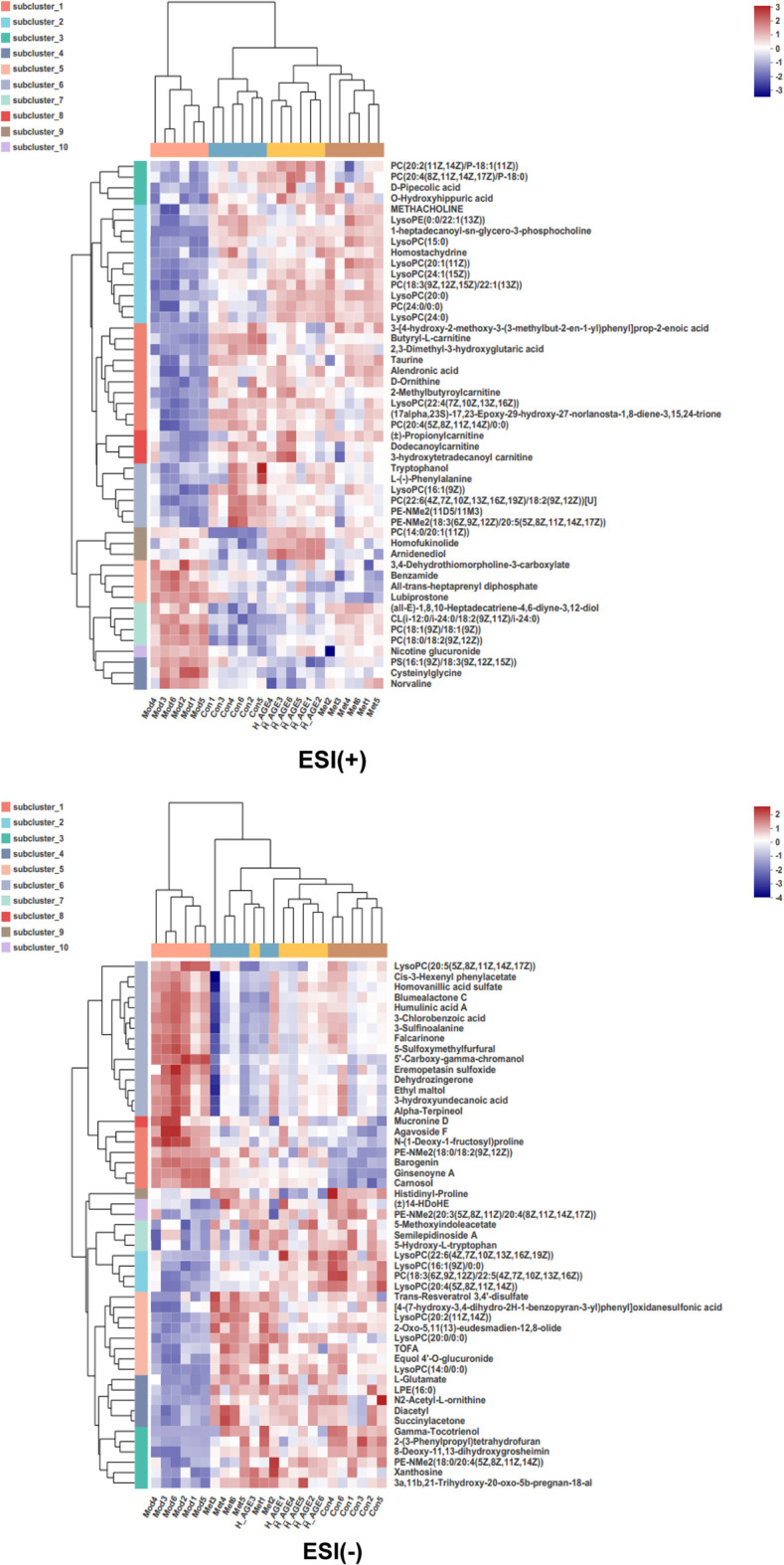
Heatmap of potential biomarkers. Rows: biomarkers; columns: samples. The shade in this picture depicts the relative expression size of metabolites in each group sample. Red depicts that the expression of biomarker was increased, blue depicts that the expression of biomarker was decreased (Mod vs. Con; Met vs. Mod; H_AGE vs. Mod).

With a threshold of VIP >1.0, *p* < 0.05, we identified 204 differential metabolites (model vs. control), 224 differential metabolites (metformin vs. model), 173 differential metabolites (L_AGE vs. model), and 191 differential metabolites (H_AGE vs. model). A total of 111 intersectant metabolites were obtained by overlapping the differential metabolites between model vs. control and metformin vs. model groups. A total of 106 intersectant metabolites were screened by overlapping the differential metabolites between model vs. control and H_AGE vs. model groups. Among these 106 metabolites, 101 significant differential metabolites can be searched by HMDB or KEGG databases and were viewed as potential biomarkers of AGE against T2DM (Supplementary Table S1). Compared with the model group, the expression of 94 potential biomarkers was significantly backregulated in the H_AGE group. Figure 6 intuitively presents the relative expression of 101 potential biomarkers (49 from the ESI + mode and 52 from the ESI- mode) in the serum samples of control, model, H_AGE, and metformin groups in the form of a heatmap.

#### Metabolomics pathway results

**FIGURE 7:**
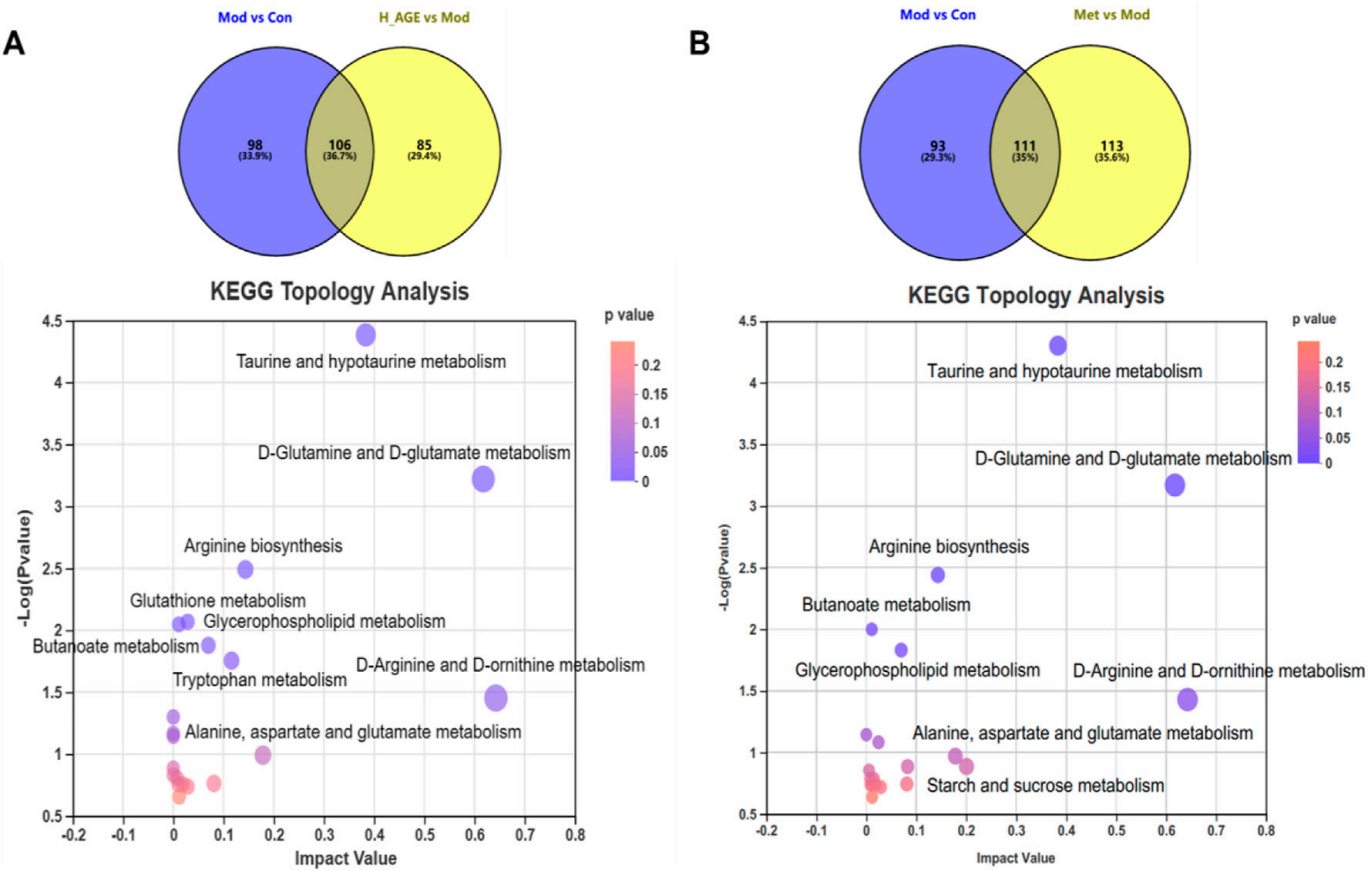
Metabolic pathway analysis. **(A)** refers to the pathway enrichment analysis of the intersectant metabolites between Mod vs Con and H_AGE vs Mod. **(B)** refers to the pathway enrichment analysis of the intersectant metabolites between Mod vs Con and Met vs Mod. The X-axis represents the impact value and the Y-axis represents–log10(*p*-value). The size of the bubble represents the impact value. The larger the bubble and higher the–log10(*p*-value), the greater the importance of the pathway.

To explore the connection among potential biomarkers, metabolic pathways, and metabolic mechanisms of AGE against T2DM, the 106 intersectant metabolites between model vs. control and H_AGE vs. model groups carried out pathway enrichment (Figure 7A). The metabolic pathways with *p* < 0.05 and impact value >0.1 were thought of as the most important pathways, including D-glutamine and D-glutamate metabolism, taurine and hypotaurine metabolism, arginine biosynthesis, tryptophan metabolism, and D-arginine and D-ornithine metabolism (Table 1). Metabolites enriched in these important pathways were probably the most important potential biomarkers, including l-glutamate, taurine, 3-sulfinoalanine, 5-hydroxy-l-tryptophan, 5-methoxyindoleacetate, and D-ornithine . To further explore the differences in the metabolic pathways and mechanisms of AGE and metformin in treating T2DM, we also performed pathway enrichment analysis on 111 intersectant metabolites between model vs. control and metformin vs. model groups (Figure 7B). Notably, unlike metformin, AGE also exerts its anti-T2DM effect by improving disorders of tryptophan metabolism and glutathione metabolism.

**TABLE 1:** Metabolic pathways associated with AGE treatment.

Pathway description	Impact value	*p*-value	Related metabolite
Taurine and hypotaurine metabolism	0.38	0.000	Taurine; L-glutamate; 3-sulfinoalanine
D-Glutamine and D-glutamate metabolism	0.62	0.001	L-Glutamate
Arginine biosynthesis	0.14	0.003	L-Glutamate; N2-acetyl-L-ornithine
Glutathione metabolism	0.03	0.009	Cysteinyl-glycine; L-glutamate
Butanoate metabolism	0.01	0.009	L-Glutamate; diacetyl
Glycerophospholipid metabolism	0.07	0.01	LysoPCs; PCs
Tryptophan metabolism	0.12	0.02	5-Hydroxy-l-tryptophan; 5-methoxyindoleacetate
D-Arginine and D-ornithine metabolism	0.64	0.04	D-Ornithine

## Discussion

T2DM is a metabolic syndrome with complex pathogenesis, which primarily results in the disorders of carbohydrate, lipid, and protein metabolism (DeFronzo et al., 2015). Currently, many biologically active traditional Chinese herbal medicines (TCHMs) are widely used in the treatment of type 2 diabetes and are expected to be its alternative therapies (Hasanpour et al., 2020). Metabolomics, as an important component of system biology, helps to confirm the safety and efficacy of these TCHMs, explores their potential mechanisms of action and potential biomarkers, and further identifies the targets of these TCHMs (Shi et al., 2016). Furthermore, metabolomics can unveil metabolic characteristics of the whole system after interventions, which conform to the overall notion of TCHMs and is conducive to its modern research (Wang et al., 2015). Therefore, we attempted to study the metabolic mechanisms of AGE against T2DM by metabolomics technology.

In this study, based on the pharmacodynamic evaluation results, we found that AGE can exert its significant efficacy by improving blood lipid levels and protecting the islets in T2DM rats. The metabolomics results revealed that H_AGE can greatly reverse the metabolic disorder in T2DM rats by regulating 101 potential biomarkers. Furthermore, the pathway enrichment and KEGG topology analysis suggested that anti-T2DM mechanisms of AGE were closely associated with regulating the disorders of amino acid and lipid metabolism. Herein, we aimed to explore the potential mechanisms of AGE against T2DM by investigating some special potential biomarkers and metabolic pathways.

A previous study has shown that American ginseng can increase the content of carnitine and help restore disorders of lipid metabolism in the body (Zhang et al., 2019). However, it was found that butyryl-L-carnitine (BC), as a kind of butyrate ester of carnitine, can produce butyrate and carnitine and is mainly used to alleviate gut inflammation through carnitine transporter OCTN2 and the amino acid transporter ATB^0,+^ (Srinivas et al., 2007). In addition, propionylcarnitine (PC), is a kind of analog of L-carnitine, can alleviate peripheral vascular disease–related (PAD-related) symptoms and decrease glycemic levels in T2DM patients with PAD (Ragozzino et al., 2004). An experiment also has revealed that PC can improve cardiac function and enhance ischemic tolerance of the diabetic rat heart (Broderick et al., 2004). The expression of BC and PC was upregulated after treatment, indicating that AGE and metformin are beneficial for alleviating inflammation, and improving heart function in diabetic rats. Gamma-tocotrienol is an isomer of unsaturated vitamin E, which can alleviate adipose tissue inflammation and maintain insulin sensitivity by suppressing nuclear factor kappa-B (NF-κB) and Nod-like receptor 3 (NLRP3) inflammasome (Kim et al., 2016). Compared to the model group, the expression of gamma-tocotrienol was upregulated in the H_AGE and metformin group, demonstrating that the anti-T2DM effect of H_AGE and metformin may be closely linked to inflammation.

The metabonomics data presented that the contents of lysophosphatidylcholines (lysoPCs), lysophosphatidylethanolamines (lysoPEs), and phosphatidylcholines (PCs) expect for lysoPC(20:5 (5Z,8Z,11Z,14Z,17Z)), PC(18:1 (9Z)/18:1 (9Z)),PC(14:0/20:1 (11Z)), and PC(18:0/18:2 (9Z,12Z) were downregulated in model rats and upregulated in AGE rats, suggesting that lipid metabolism may play an essential role in AGE against T2DM. A research study also manifested that these lipid levels were downregulated in T2DM patients with cardiovascular disease (CVD) compared with the T2DM patients, indicating that the expression of these lipids is negatively correlated with the risk of cardiovascular disease in T2DM patients (Garcia-Fontana et al., 2016). Moreover, previous studies have shown that lysoPCs and lysoPEs can be used as circulating metabolic intermediates associated with inflammation and oxidative stress to predict the risk of early diabetes (Ha et al., 2012). A previous report revealed that lysoPC can play an important role in modulating vascular endothelial dilation, cellular immune response, and insulin secretion (Yamamoto et al., 2021). Phosphatidylcholine (PC) is the most vital phospholipid enriched in the endoplasmic reticulum, which is involved in lipid storage/secretion, stress response, and the occurrence of diabetes (Lagace and Ridgway, 2013; Wang et al. (2011) suggested that choline, trimethylamine N-oxide (TMAO), and betaine, as the three metabolites of the dietary lipid phosphatidylcholine, were helpful to predict risk for cardiovascular diseases (CVDs).

Trans-resveratrol 3,4′-disulfate belongs to the class of organic compounds known as stilbenes. Stilbenes are important polyphenolic compounds with a variety of pharmacological activities such as anti-inflammatory, anti-diabetic, and anti-lipidemic and have significant modulating effects on angiogenesis, inflammation, and cell proliferation (Tsai et al., 2017; ShazmeenHaq et al., 2021). The expression of trans-resveratrol 3,4′-disulfate was significantly increased after both H_AGE and metformin treatment, indicating that this compound may play an essential role in AGE and metformin against T2DM.

Tryptophan metabolism is an important metabolic pathway involved in regulating inflammation-related diseases. l-Tryptophan (tryptophan) can decrease blood glucose levels and preserve normal insulin secretion in T2DM rats (Sorgdrager et al., 2019). 5-Hydroxy-l-tryptophan (5-HTP), as the immediate precursor in the biosynthesis of 5-hydroxy-tryptamine (5-HT), can inhibit insulin and glucagon secretion in non-diabetic islet donors and significantly increased the release of insulin in response to glucose in islets isolated from T2D donors (Bennet et al., 2015, (Das et al., 2004). In our study, 5-HTP enriched in the tryptophan metabolism pathway was downregulated in the model rats and upregulated in the H_AGE rats, demonstrating that H_AGE may promote insulin secretion by regulating tryptophan metabolism. Glutathione (γ-glutamyl-cysteine glycine) belongs to a class of low molecular weight thiols synthesized from glutamate, cysteine, and glycine (Wu et al., 2004). Glutathione deficiency is highly likely to lead to the development of T2DM (Hakki Kalkan and Suher, 2013). Glutathione metabolism plays a key role in inhibiting oxidative stress and cysteinyl-glycine can be decomposed into cysteine and glycine under the catalysis of dipeptidase (Lutchmansingh et al., 2018). Our study found that cysteinyl-glycine and l-glutamate were enriched in the glutathione metabolic pathway. The lower cysteinyl-glycine level and higher l-glutamate level in the H_AGE group compared to the model group, indicate that AGE may further synthesize glutathione by promoting the production of cysteine, glycine, and l-glutamate to suppress the process of T2DM.

L-Arginine (arginine), as the substrate for NO production by endothelial cells, is effective in the treatment of endothelial dysfunction triggered by cardiovascular risk factors (Gambardella et al., 2020). It can stimulate insulin production by acting on voltage-gated Ca^2+^ channels and promote insulin secretion through transforming to l-glutamate (Newsholme and Krause, 2012). l-Glutamate, as a kind of non-essential amino acid abundant in the body and a substrate in metabolism, is derived from the biological synthesis and food intake, as well as enhances insulin secretion (Takahashi et al., 2019). Han, et al. (2021) said that glutamate converted from glutamine is an essential mediator that enhances calcium signaling in the glutamine-amplifying effect on insulin secretion. We observed that the l-glutamate content was downregulated in the model rats and was significantly upregulated after H_AGE and metformin treatment, suggesting that AGE may stimulate insulin secretion by regulating multiple metabolic pathways related to l-glutamate.

Taurine is a type of abundant amino acid that existed in all mammalian tissues, which can improve the disorders of glucose metabolism and lipid metabolism by decreasing serum leptin levels and insulin resistance (Kim et al., 2012). Increasing evidence has shown that taurine can play a beneficial effect on diabetes and its complications through the suppression of oxidative stress and inflammation (Ito et al., 2012; Qaradakhi et al., 2020). Taurine levels increased after H_AGE and metformin treatment, suggesting that H_AGE exerts its efficacy by interfering with the metabolism of taurine and hypotaurine, which is consistent with the past research study (Zhang et al., 2019). Ornithine is an intermediate compound produced by the enzymatic action of arginase and synthesized into urea through the urea cycle (Sivashanmugam and Jaidev 2017). A hospital-based cross-sectional study revealed a negative correlation between T2DM and ornithine levels in the urea cycle, which is consistent with our present results (Cao et al., 2019). A study showed that amino acids involved in the urea cycle are associated with inflammation and oxidative stress (Carracedo et al., 2011). These facts indicated that H_AGE and metformin may mainly exert anti-inflammatory and anti-oxidant effects through the regulation of amino acid metabolism.

TOFA (5-(tetradecyloxy)-2-furoic acid) is an effective hypolipidemic agent that can significantly inhibit fatty acid synthesis, stimulate fatty acid oxidation, and reduce triglyceride synthesis (Halvorson and McCune, 1984; Fukuda and Ontko, 1984). In addition, it was reported that a-terpineol can increase lipid levels by modulating the AMPK/mTOR/SREBP-1 pathway. Compared with the model group, H_AGE and metformin can significantly increase the content of TOFA and decrease the content of a-terpineol, indicating that H_AGE and metformin may exert their anti-T2DM effect by reducing lipid levels. Pipecolic acid is a non-protein amino acid originating from lysine catabolism and is essential for the regulation of human and plant immunity (Wang et al., 2018). According to previous studies, pipecolic acid acts as a key regulator of various plant defense responses and contributes to immune signaling salicylic acid (SA) production, antimicrobial metabolite accumulation, and transcription of defense genes (Zeier, 2013). The higher level of D-pipecolic acid in the H_AGE and metformin rats indicated that AGE and metformin may enhance body immune response by increasing the content of D-pipecolic acid.

## Conclusion

Ultimately, the curative effect of AGE and metformin on T2DM was verified by biochemical analysis and pancreas section analysis. Based on UHPLC–MS/MS–based untargeted metabolomics technology and multivariate statistical analysis, we found that AGE can have a significant anti-T2DM effect by modulating 94 potential biomarkers contents. The enrichment analysis revealed that AGE mainly had an impact on the metabolic disturbance of D-glutamine and D-glutamate metabolism, taurine, and hypotaurine metabolism, arginine biosynthesis, and tryptophan metabolism, and D-arginine and D-ornithine metabolism. Further analysis demonstrated that the anti-T2DM mechanisms of AGE were mainly related to suppressing inflammation, oxidative stress, endothelial dysfunction, dyslipidemia, insulin resistance, T2DM-related complications and enhancing immune response, and insulin secretion. Our study systematically elucidated for the first time that the potential biomarkers and mechanisms of AGE against T2DM from a metabolic perspective, which can provide a reference for new drug development and the clinical diagnosis of T2DM.

## Data Availability

The original contributions presented in the study are included in the article/Supplementary Material; further inquiries can be directed to the corresponding authors.
