# Supplementary material for: Study on the mechanism of American ginseng extract for treating type 2 diabetes mellitus based on metabolomics
Source: Front Pharmacol. 2022 Sep 2;13:960050. doi: 10.3389/fphar.2022.960050 (PMC9479495; doi:10.3389/fphar.2022.960050)
Supplement: Supplementary file 1 [file DataSheet1.doc]

**A**


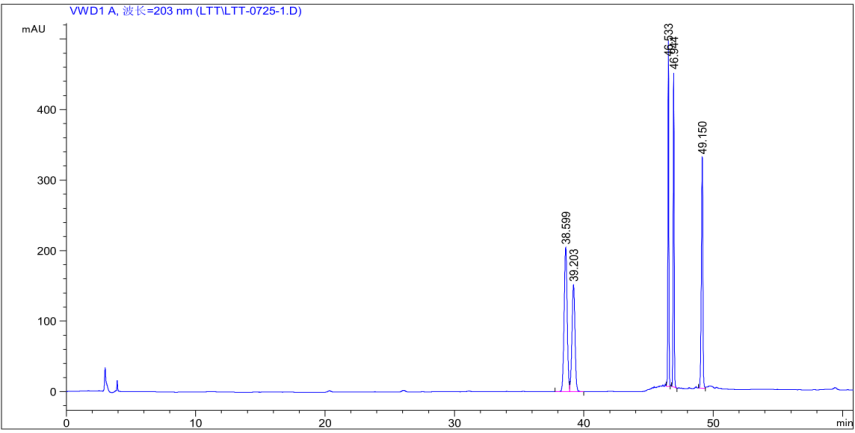


**B**


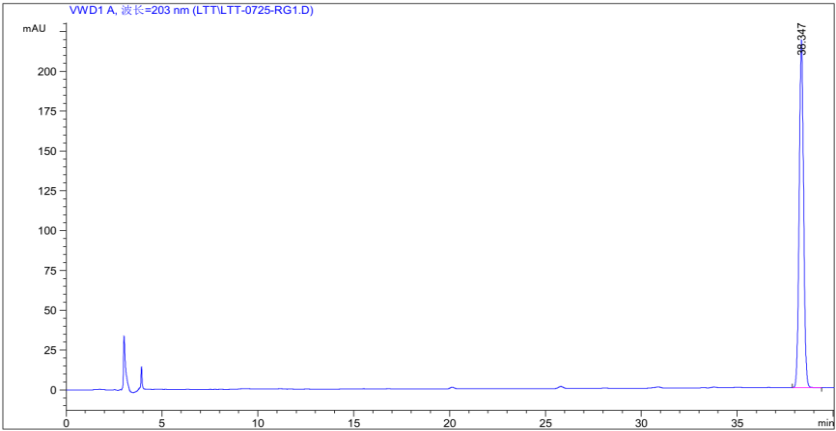


**C**


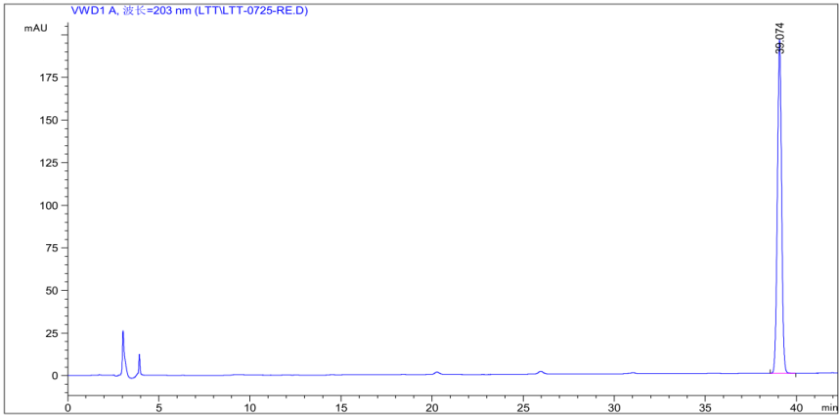


C


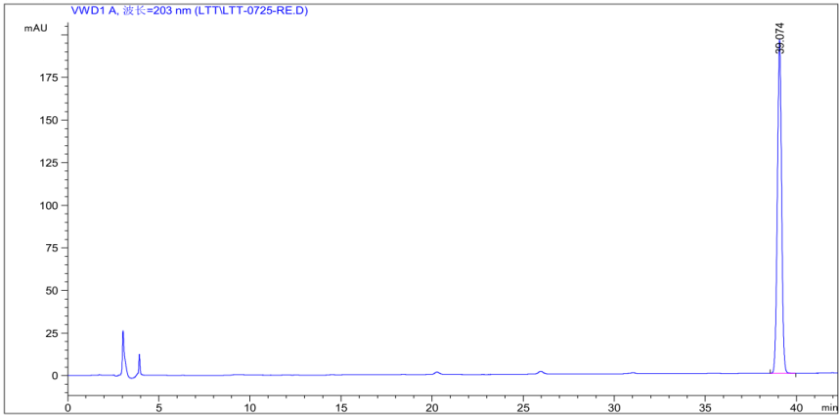


C


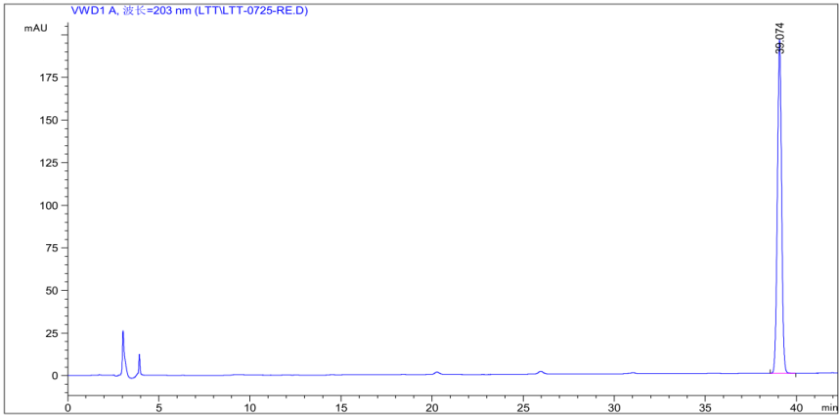


C


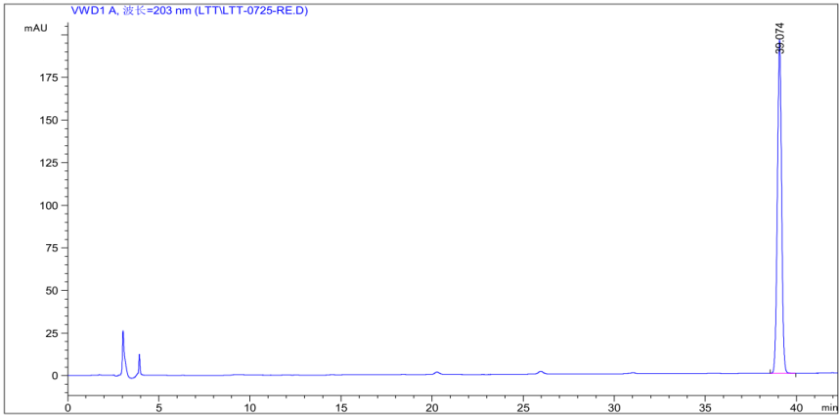


C


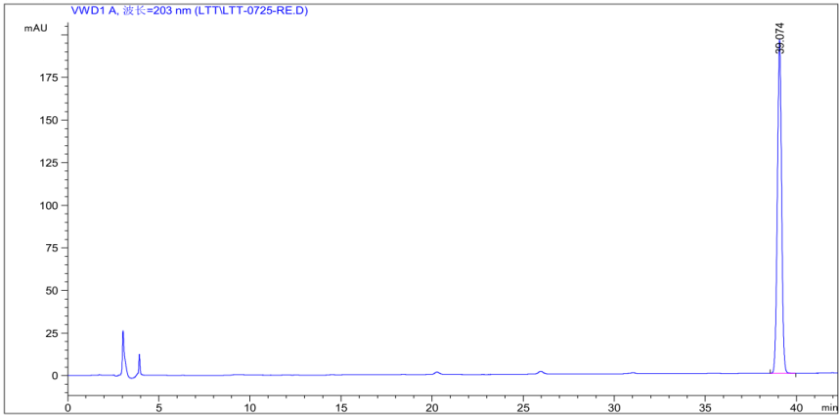


C


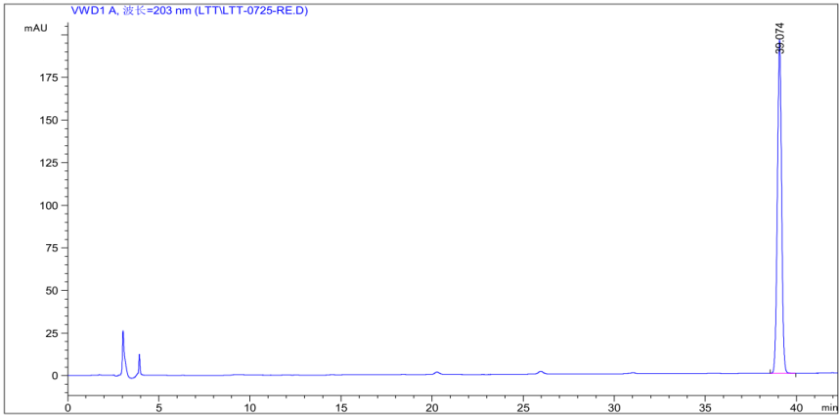


**D**


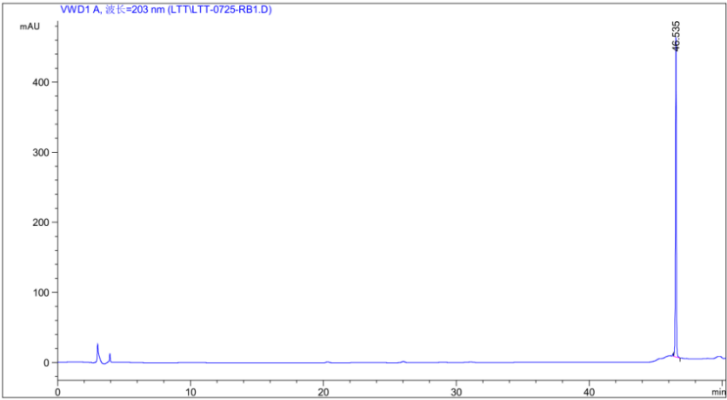


**E**


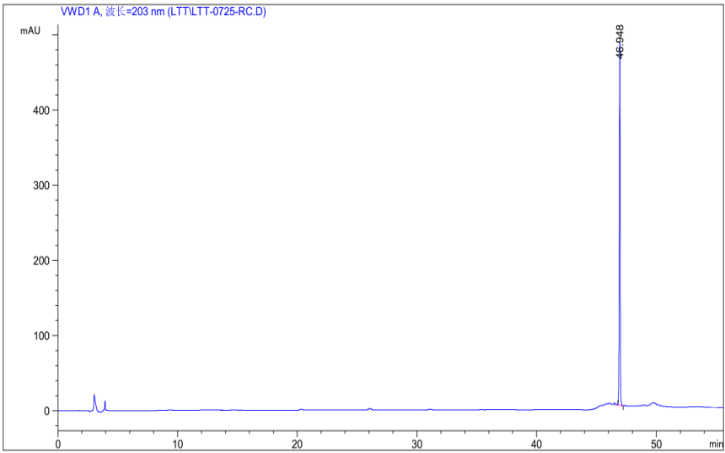


**F**


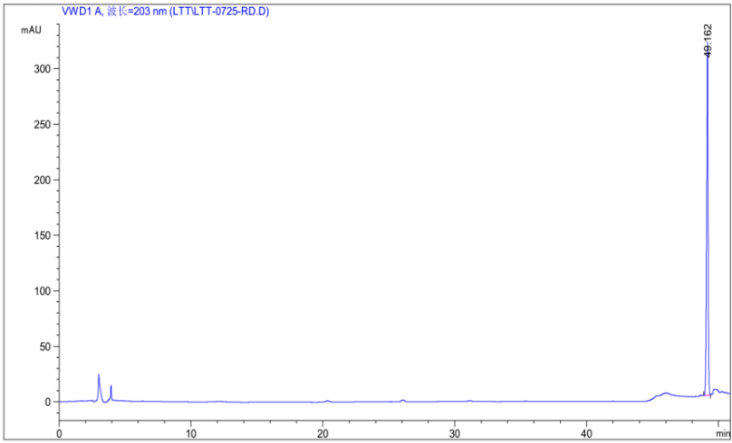

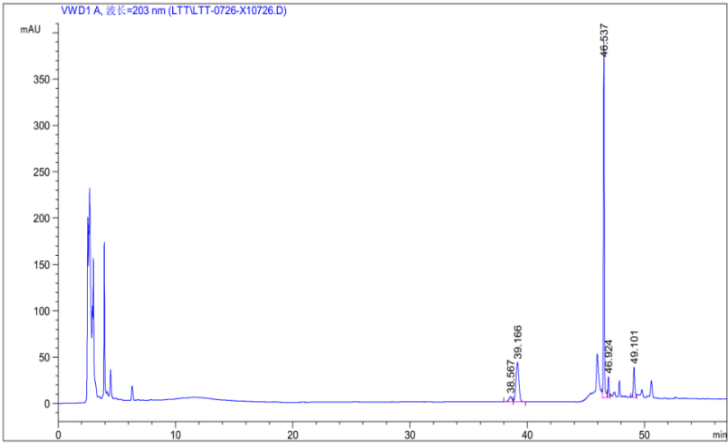


G


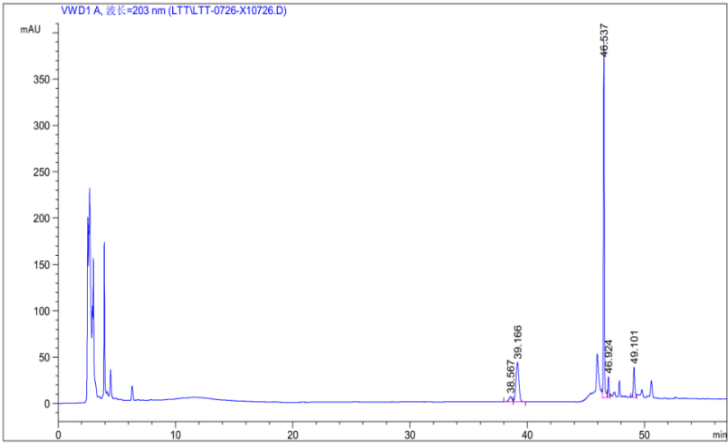


G


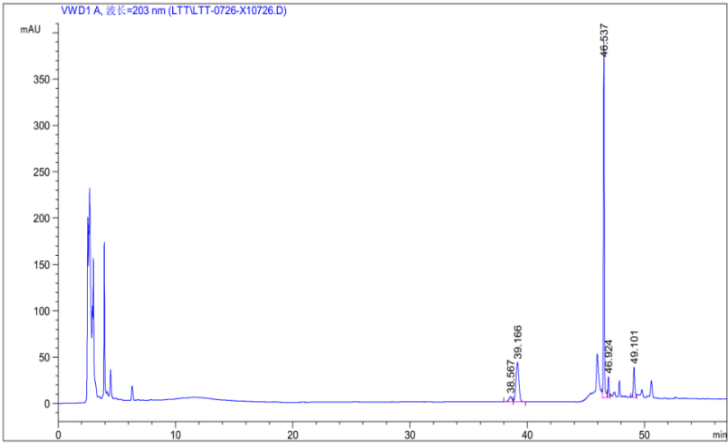


**G**

**Supplementary Figure S1** The corresponding chromatogram. Note: **A** represents mixed standard; **B, C, D, E,** and **F** respectively present ginsenoside Rg1, Re, Rb1, Rc, and Rd; **G** represents AGE.

**
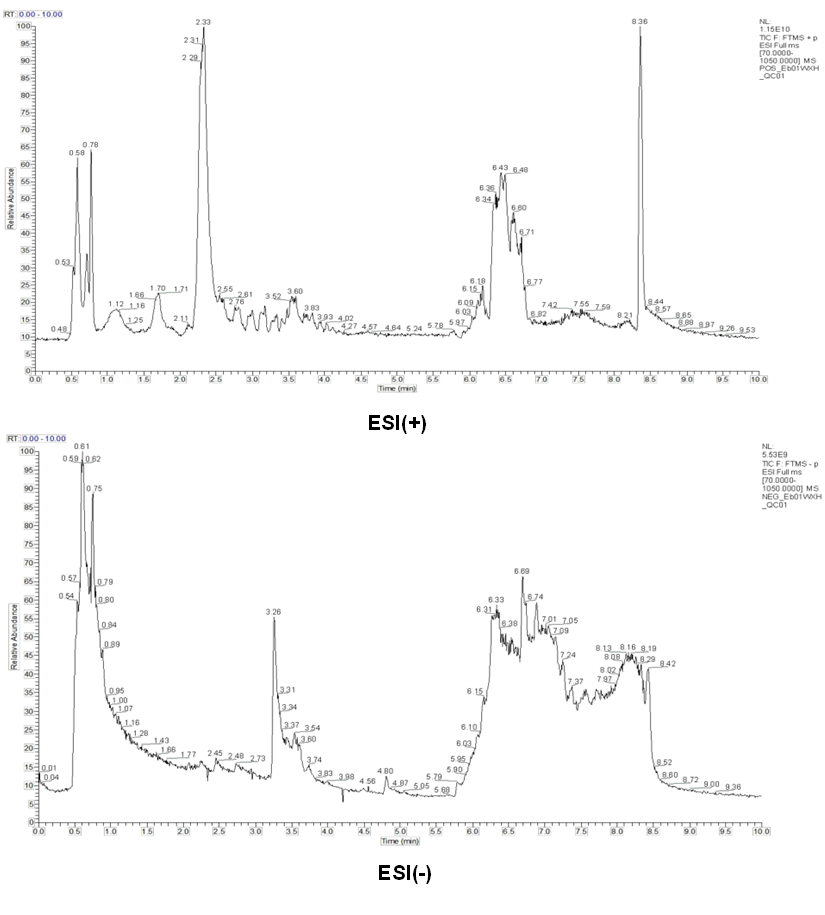
**

**Supplementary Figure S2** The total ion chromatograms (TIC) of sample QC01.


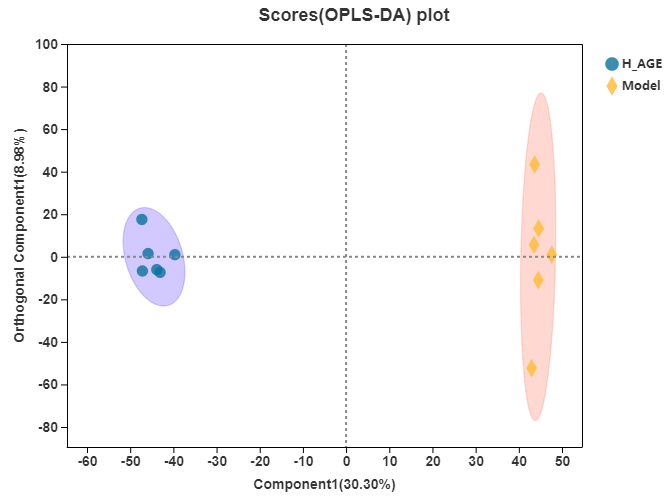

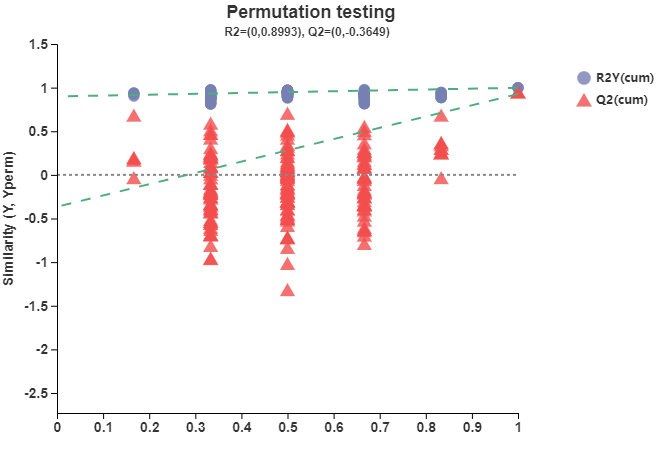

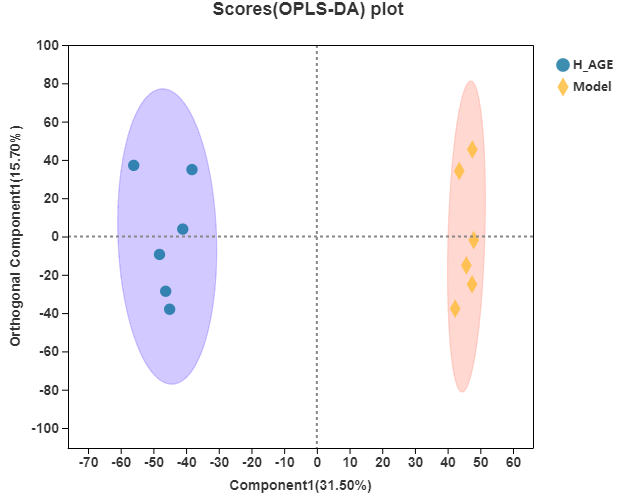

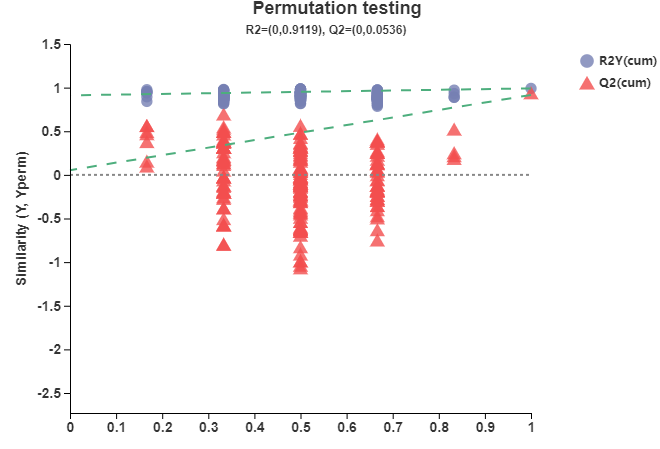


**A**

**B**


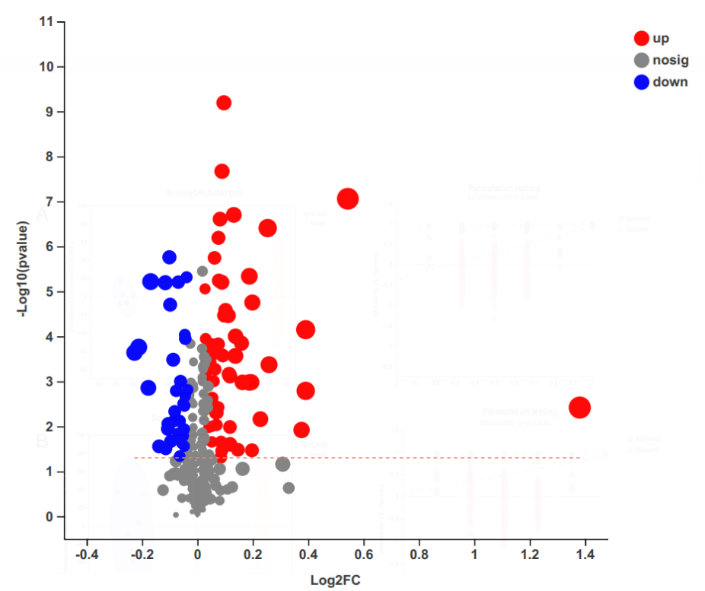

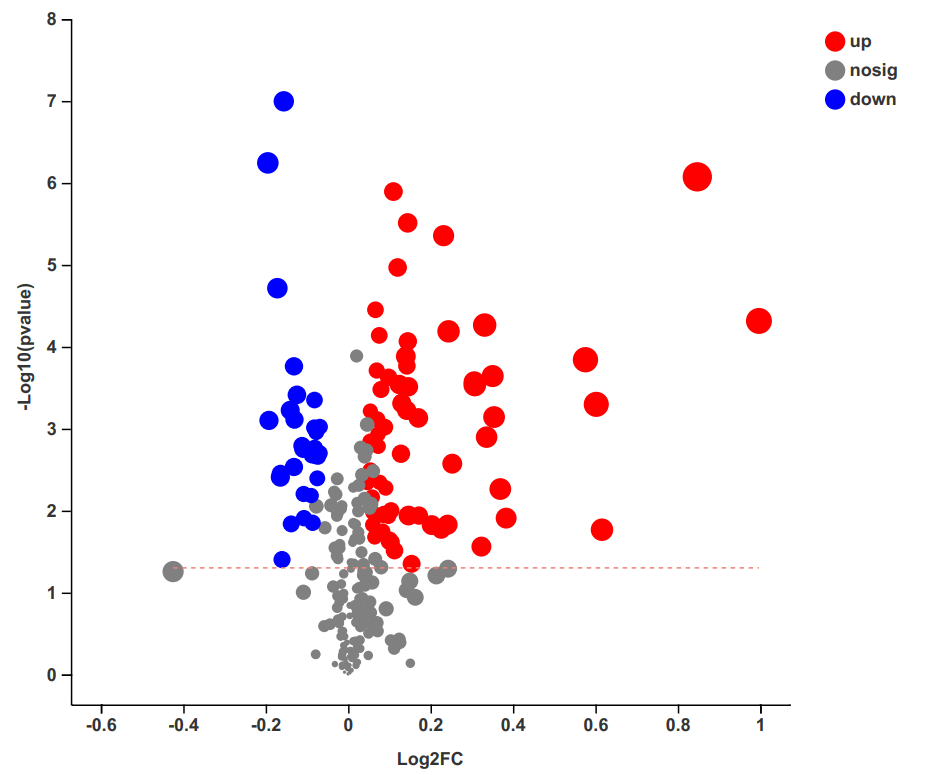


**C**

**D**

**E**

**F**

**Supplementary Figure S3** OPLS-DA score diagrams of the samples from H_AGE vs Model in the ESI+ (**A**) or ESI- mode (**B**); the 7-fold cross validation plot of OPLS-DA model with 200 permutation tests in the positive mode **(C)** or negative mode **(D)**; the volcano plots of the samples from H_AGE vs Model in the ESI+ (**E**) or ESI- mode (**F**).
