# Supplementary material for: Study on the mechanism of American ginseng extract for treating type 2 diabetes mellitus based on metabolomics
Source: Front Pharmacol. 2022 Sep 2;13:960050. doi: 10.3389/fphar.2022.960050 (PMC9479495; doi:10.3389/fphar.2022.960050)
Supplement: Supplementary file 2 [file Table1.doc]

**Supplementary Table 1** The potential biomarkers linked to H_AGE treatment.

| **mode** | **Metabolite** | **Formula** | **M/Z** | **Library ID** | **FC _M_** | **FC _H_** | **ModvsCon** | **H_AGEvsMod** |
| --- | --- | --- | --- | --- | --- | --- | --- | --- |
| neg | N-(1-Deoxy-1-fructosyl)proline | C_11_H_19_NO_7_ | 276.11 | HMDB0038493 | 1.06 | 0.95 | ↑*** | ↓^##^ |
| neg | Equol 4'-O-glucuronide | C_21_H_22_O_9_ | 417.12 | HMDB0041731 | 0.84 | 1.18 | ↓*** | ↑^##^ |
| neg | [4-(7-hydroxy-3，4-dihydro-2H-1-benzopyran-3-yl)phenyl]oxidanesulfonic acid | C_15_H_14_O_6_S | 321.04 | HMDB0141213 | 0.89 | 1.11 | ↓* | ↑^#^ |
| neg | L-Glutamate | C_5_H_9_NO_4_ | 146.04 | - | 0.97 | 1.04 | ↓** | ↑^##^ |
| neg | 5'-Carboxy-gamma-chromanol | C_18_H_26_O_4_ | 305.18 | HMDB0012799 | 1.13 | 0.90 | ↓*** | ↓^##^ |
| neg | Carnosol | C_20_H_26_O_4_ | 329.18 | HMDB0002121 | 1.49 | 0.89 | ↓*** | ↓^##^ |
| neg | Barogenin | C_27_H_42_O_4_ | 429.30 | HMDB0034403 | 1.21 | 0.91 | ↓*** | ↓^##^ |
| neg | LysoPC(22:6(4Z，7Z，10Z，13Z，16Z，19Z)) | C_30_H_50_NO_7_P | 612.33 | HMDB0010404 | 0.97 | 1.03 | ↓** | ↑^##^ |
| neg | Alpha-Terpineol | C_10_H_18_O | 199.13 | HMDB0004043 | 1.05 | 0.95 | ↓** | ↓^##^ |
| neg | LysoPC(20:4(5Z，8Z，11Z，14Z)) | C_28_H_50_NO_7_P | 588.33 | HMDB0010395 | 0.95 | 1.04 | ↓*** | ↑^##^ |
| neg | LPE(16:0) | C_21_H_44_NO_7_P | 498.29 | HMDB0011503 | 0.82 | 1.24 | ↓*** | ↑^##^ |
| neg | 5-Hydroxy-L-tryptophan | C_11_H_12_N_2_O_3_ | 219.08 | HMDB0000472 | 0.92 | 1.07 | ↓** | ↑^##^ |
| neg | Semilepidinoside A | C_16_H_20_N_2_O_6_ | 381.13 | HMDB0033107 | 0.94 | 1.08 | ↓* | ↑^#^ |
| neg | Succinylacetone | C_7_H_10_O_4_ | 203.06 | HMDB0000635 | 0.83 | 1.24 | ↓** | ↑^##^ |
| neg | Trans-Resveratrol 3，4'-disulfate | C_14_H_12_O_9_S_2_ | 368.97 | HMDB0041780 | 0.62 | 1.53 | ↓* | ↑^#^ |
| neg | 5-Methoxyindoleacetate | C_11_H_11_NO_3_ | 204.07 | HMDB0004096 | 0.90 | 1.13 | ↓** | ↑^#^ |
| neg | 4-Hydroperoxy-2-nonenal | C_9_H_16_O_3_ | 217.11 | HMDB0060287 | 0.95 | 0.94 | ↓* | ↓^##^ |
| neg | 5-Sulfoxymethylfurfural | C_6_H_6_O_6_S | 250.99 | HMDB0059752 | 1.04 | 0.95 | ↓** | ↓^##^ |
| neg | Eremopetasin sulfoxide | C_19_H_26_O_4_S | 331.14 | HMDB0032003 | 1.13 | 0.88 | ↓** | ↓^##^ |
| neg | Ethyl maltol | C_7_H_8_O_3_ | 185.04 | HMDB0031735 | 1.06 | 0.94 | ↓* | ↓^##^ |
| neg | 3-Chlorobenzoic acid | C_7_H_5_ClO_2_ | 154.99 | HMDB0001544 | 1.04 | 0.95 | ↓** | ↓^##^ |
| neg | 2-(3-Phenylpropyl)tetrahydrofuran | C_13_H_18_O | 235.13 | HMDB0036178 | 0.87 | 1.05 | ↓*** | ↑^##^ |
| neg | 3a，11b，21-Trihydroxy-20-oxo-5b-pregnan-18-al | C_21_H_32_O_5_ | 363.22 | HMDB0006753 | 0.83 | 1.28 | ↓** | ↑^##^ |
| neg | Ginsenoyne A | C_17_H_22_O_2_ | 303.16 | HMDB0039107 | 1.42 | 0.87 | ↓*** | ↓^###^ |
| neg | LysoPC(20:5(5Z，8Z，11Z，14Z，17Z)) | C_28_H_48_NO_7_P | 586.31 | HMDB0010397 | 1.04 | 0.92 | ↓* | ↓^###^ |
| neg | 14-HDoHE | C_22_H_32_O_3_ | 343.23 | HMDB0060044 | 0.96 | 1.04 | ↓* | ↑^#^ |
| neg | Humulinic acid A | C_15_H_22_O_4_ | 287.13 | HMDB0030104 | 1.06 | 0.93 | ↓*** | ↓^##^ |
| neg | TOFA | C_19_H_32_O_4_ | 323.22 | - | 0.84 | 1.18 | ↓*** | ↑^##^ |
| neg | PE-NMe2(20:3(5Z，8Z，11Z)/20:4(8Z，11Z，14Z，17Z)) | C_47_H_80_NO_8_P | 862.56 | HMDB0114286 | 0.92 | 1.06 | ↓** | ↑^#^ |
| neg | Mucronine D | C_37_H_51_N_5_O_6_ | 642.36 | HMDB0029335 | 1.08 | 0.94 | ↓** | ↓^#^ |
| neg | PC(18:3(6Z，9Z，12Z)/22:5(4Z，7Z，10Z，13Z，16Z)) | C_48_H_80_NO_8_P | 874.56 | HMDB0008187 | 0.93 | 1.06 | ↓*** | ↑^###^ |
| neg | PE-NMe2(18:0/18:2(9Z，12Z)) | C_43_H_82_NO_8_P | 770.57 | HMDB0113990 | 1.40 | 0.89 | ↓*** | ↓^#^ |
| neg | PE-NMe2(18:0/20:4(5Z，8Z，11Z，14Z)) | C_45_H_82_NO_8_P | 794.57 | HMDB0113999 | 0.94 | 1.06 | ↓*** | ↑^###^ |
| neg | LysoPC(20:0/0:0) | C_28_H_58_NO_7_P | 596.39 | HMDB0010390 | 0.96 | 1.08 | ↓** | ↑^###^ |
| neg | LysoPC(20:2(11Z，14Z)) | C_28_H_54_NO_7_P | 592.36 | HMDB0010392 | 0.94 | 1.05 | ↓*** | ↑^###^ |
| neg | Falcarinone | C_17_H_22_O | 287.17 | HMDB0033693 | 1.05 | 0.93 | ↓* | ↓^##^ |
| neg | Cis-3-Hexenyl phenylacetate | C_14_H_18_O_2_ | 253.10 | HMDB0038281 | 1.05 | 0.91 | ↓* | ↓^###^ |
| neg | LysoPC(14:0/0:0) | C_22_H_46_NO_7_P | 466.29 | HMDB0010379 | 0.91 | 1.11 | ↓*** | ↑^###^ |
| neg | 3-Sulfinoalanine | C_3_H_6_NO_4_S | 188.95 | HMDB0000996 | 1.04 | 0.94 | ↓** | ↓^###^ |
| neg | LysoPC(16:1(9Z)/0:0) | C_24_H_48_NO_7_P | 538.31 | HMDB0010383 | 0.94 | 1.05 | ↓*** | ↑^###^ |
| neg | Blumealactone C | C_17_H_24_O_6_ | 345.13 | HMDB0035357 | 1.07 | 0.91 | ↓*** | ↓^###^ |
| neg | Gamma-Tocotrienol | C_28_H_42_O_2_ | 455.32 | HMDB0012958 | 0.65 | 1.26 | ↓*** | ↑^##^ |
| neg | 3-hydroxyundecanoic acid | C_11_H_22_O_3_ | 201.15 | HMDB0061654 | 1.05 | 0.95 | ↓** | ↓^##^ |
| neg | Homovanillic acid sulfate | C_9_H_10_O_7_S | 296.99 | HMDB0011719 | 1.07 | 0.93 | ↓** | ↓^#^ |
| neg | 8-Deoxy-11，13-dihydroxygrosheimin | C_15_H_20_O_5_ | 279.12 | HMDB0041144 | 0.73 | 1.26 | ↓*** | ↑^###^ |
| neg | 2-Oxo-5，11(13)-eudesmadien-12，8-olide | C_15_H_18_O_3_ | 291.12 | HMDB0030911 | 0.86 | 1.09 | ↓*** | ↑^###^ |
| neg | Dehydrozingerone | C_11_H_12_O_3_ | 237.08 | HMDB0032591 | 1.09 | 0.91 | ↓*** | ↓^###^ |
| neg | Agavoside F | C_68_H_110_O_35_ | 742.33 | HMDB0032940 | 1.27 | 0.89 | ↓*** | ↓^##^ |
| neg | Diacetyl | C_4_H_6_O_2_ | 217.07 | HMDB0003407 | 0.81 | 1.28 | ↓** | ↑^###^ |
| neg | Histidinyl-Proline | C_11_H_16_N_4_O_3_ | 233.10 | HMDB0028893 | 0.89 | 0.89 | ↓*** | ↓^##^ |
| neg | Xanthosine | C_10_H_12_N_4_O_6_ | 283.07 | HMDB0000299 | 0.92 | 1.13 | ↓* | ↑^###^ |
| neg | N2-Acetyl-L-ornithine | C_7_H_14_N_2_O_3_ | 173.09 | - | 0.92 | 1.09 | ↓** | ↑^###^ |
| pos | Arnidenediol | C_30_H_50_O_2_ | 407.37 | HMDB0035401 | 1.17 | 1.46 | ↑ * | ↑^###^ |
| pos | PC(20:4(5Z，8Z，11Z，14Z)/0:0) | C_28_H_50_NO_7_P | 544.34 | LMGP01050048 | 0.97 | 1.02 | ↓*** | ↑^###^ |
| pos | Nicotine glucuronide | C_16_H_22_N_2_O_6_ | 339.15 | HMDB0001272 | 1.06 | 0.97 | ↓*** | ↓^#^ |
| pos | LysoPE(0:0/22:1(13Z)) | C_27_H_54_NO_7_P | 558.35 | HMDB0011491 | 0.93 | 1.04 | ↓*** | ↑^###^ |
| pos | LysoPC(20:0) | C_28_H_58_NO_7_P | 574.38 | - | 0.98 | 1.06 | ↓** | ↑^###^ |
| pos | PC(20:4(8Z，11Z，14Z，17Z)/P-18:0) | C_46_H_84_NO_7_P | 816.59 | HMDB0008489 | 0.98 | 1.03 | ↓** | ↑^###^ |
| pos | PC(22:6(4Z，7Z，10Z，13Z，16Z，19Z)/18:2(9Z，12Z)) | C_48_H_80_NO_8_P | 830.56 | HMDB0008730 | 0.97 | 1.02 | ↓*** | ↑^###^ |
| pos | LysoPC(16:1(9Z)) | C_24_H_48_NO_7_P | 494.32 | - | 0.96 | 1.03 | ↓*** | ↑^###^ |
| pos | L-Phenylalanine | C_9_H_11_NO^2^ | 188.07 | HMDB0000159 | 0.91 | 1.08 | ↓* | ↑^###^ |
| pos | Lubiprostone | C_20_H_32_F_2_O_5_ | 413.21 | HMDB0015180 | 1.06 | 0.93 | ↓** | ↓^###^ |
| pos | Taurine | C_2_H_7_NO_3_S | 126.02 | HMDB0000251 | 0.94 | 1.05 | ↓*** | ↑^##^ |
| pos | Homofukinolide | C_25_H_34_O_6_ | 469.20 | HMDB0034659 | 1.09 | 1.09 | ↑* | ↑^#^ |
| pos | 3，4-Dehydrothiomorpholine-3-carboxylate | C_5_H_7_NO_2_S | 146.03 | - | 1.13 | 0.91 | ↓** | ↓^#^ |
| pos | Propionylcarnitine | C_10_H_19_NO_4_ | 218.14 | HMDB0000824 | 0.97 | 1.05 | ↓* | ↑^##^ |
| pos | Cysteinylglycine | C_5_H_10_N_2_O_3_S | 242.06 | HMDB0000078 | 1.03 | 0.97 | ↓*** | ↓^###^ |
| pos | Dodecanoylcarnitine | C_19_H_37_NO_4_ | 344.28 | HMDB0002250 | 0.94 | 1.05 | ↓*** | ↑^##^ |
| pos | 3-hydroxytetradecanoyl carnitine | C_21_H_41_NO_5_ | 370.29 | HMDB0061640 | 0.97 | 1.04 | ↓* | ↑^#^ |
| pos | LysoPC(15:0) | C_23_H_48_NO_7_P | 504.30 | HMDB0010381 | 0.95 | 1.06 | ↓*** | ↑^###^ |
| pos | LysoPC(22:4(7Z，10Z，13Z，16Z)) | C_30_H_54_NO_7_P | 572.37 | HMDB0010401 | 0.95 | 1.05 | ↓*** | ↑^###^ |
| pos | All-trans-heptaprenyl diphosphate | C_35_H_60_O_7_P_2_ | 672.42 | HMDB0012187 | 1.03 | 0.97 | ↓*** | ↓^##^ |
| pos | (all-E)-1，8，10-Heptadecatriene-4，6-diyne-3，12-diol | C_17_H_22_O_2_ | 558.36 | HMDB0039737 | 1.11 | 0.96 | ↓*** | ↓^#^ |
| pos | LysoPC(24:0) | C_32_H_66_NO_7_P | 630.45 | HMDB0010405 | 0.92 | 1.19 | ↓** | ↑^###^ |
| pos | CL(i-12:0/i-24:0/18:2(9Z，11Z)/i-24:0) | C_87_H_166_O_17_P_2_ | 795.57 | HMDB0089408 | 1.25 | 0.85 | ↓*** | ↓^###^ |
| pos | PC(18:1(9Z)/18:1(9Z)) | C_44_H_84_NO_8_P | 824.55 | HMDB0000593 | 1.06 | 0.95 | ↓*** | ↓^###^ |
| pos | PC(14:0/20:1(11Z)) | C_42_H_82_NO_8_P | 798.54 | HMDB0007879 | 1.31 | 1.05 | ↑*** | ↑** |
| pos | PC(20:2(11Z，14Z)/P-18:1(11Z)) | C_46_H_86_NO_7_P | 818.60 | HMDB0008359 | 0.94 | 1.14 | ↓* | ↑^###^ |
| pos | PC(18:0/18:2(9Z，12Z)) | C_44_H_84_NO_8_P | 786.60 | HMDB0008039 | 1.07 | 0.97 | ↓*** | ↓^###^ |
| pos | PC(18:3(9Z，12Z，15Z)/22:1(13Z)) | C_48_H_88_NO_8_P | 860.61 | HMDB0008217 | 0.95 | 1.07 | ↓*** | ↑^###^ |
| pos | PC(24:0/0:0) | C_32_H_66_NO_7_P | 608.46 | LMGP01050057 | 0.86 | 1.31 | ↓* | ↑^###^ |
| pos | PE-NMe2(11D5/11M3) | C_48_H_84_NO_10_P | 848.57 | HMDB0114671 | 0.95 | 1.04 | ↓*** | ↑^###^ |
| pos | LysoPC(24:1(15Z)) | C_32_H_64_NO_7_P | 628.43 | HMDB0010406 | 0.93 | 1.10 | ↓*** | ↑^###^ |
| pos | PE-NMe2(18:3(6Z，9Z，12Z)/20:5(5Z，8Z，11Z，14Z，17Z)) | C_45_H_74_NO_8_P | 820.54 | HMDB0114113 | 0.96 | 1.03 | ↓** | ↑^#^ |
| pos | PS(16:1(9Z)/18:3(9Z，12Z，15Z)) | C_40_H_7_0NO_10_P | 756.48 | HMDB0012370 | 1.06 | 0.89 | ↓*** | ↓^###^ |
| pos | LysoPC(20:1(11Z)) | C_28_H_56_NO_7_P | 550.38 | HMDB0010391 | 0.97 | 1.04 | ↓** | ↑^###^ |
| pos | 1-heptadecanoyl-sn-glycero-3-phosphocholine | C_25_H_52_NO_7_P | 510.35 | LMGP01050024 | 0.93 | 1.06 | ↓*** | ↑^###^ |
| pos | (17alpha，23S)-17，23-Epoxy-29-hydroxy-27-norlanosta-1，8-diene-3，15，24-trione | C_29_H_40_O_5_ | 507.25 | HMDB0035971 | 0.96 | 1.03 | ↓*** | ↑^###^ |
| pos | Tryptophanol | C_10_H_11_NO | 144.08 | HMDB0003447 | 0.94 | 1.04 | ↓** | ↑^##^ |
| pos | 3-[4-hydroxy-2-methoxy-3-(3-methylbut-2-en-1-yl)phenyl]prop-2-enoic acid | C_15_H_18_O_4_ | 280.15 | HMDB0124830 | 0.62 | 1.30 | ↓*** | ↑^#^ |
| pos | Benzamide | C_7_H_7_NO | 122.06 | HMDB0004461 | 1.03 | 0.96 | ↓** | ↓^###^ |
| pos | 2-Methylbutyroylcarnitine | C_12_H_23_NO_4_ | 246.17 | HMDB0000378 | 0.92 | 1.08 | ↓*** | ↑^###^ |
| pos | Butyryl-L-carnitine | C_11_H_21_NO_4_ | 232.15 | - | 0.92 | 1.04 | ↓*** | ↑^##^ |
| pos | O-Hydroxyhippuric acid | C_9_H_9_NO_4_ | 237.09 | HMDB0000840 | 0.94 | 1.08 | ↓** | ↑^###^ |
| pos | 2，3-Dimethyl-3-hydroxyglutaric acid | C_7_H_12_O_5_ | 218.10 | HMDB0002025 | 0.90 | 1.07 | ↓*** | ↑^###^ |
| pos | Norvaline | C_5_H_11_NO_2_ | 159.11 | HMDB0013716 | 1.03 | 0.95 | ↓* | ↓^##^ |
| pos | Homostachydrine | C_8_H_15_NO_2_ | 158.12 | - | 0.89 | 1.12 | ↓** | ↑^##^ |
| pos | Methacholine | C_8_H_17_NO_2_ | 160.13 | HMDB0015654 | 0.93 | 1.06 | ↓** | ↑^#^ |
| pos | Alendronic acid | C_4_H_13_NO_7_P_2_ | 214.01 | HMDB0001915 | 0.93 | 1.10 | ↓*** | ↑^###^ |
| pos | D-Pipecolic acid | C_6_H_11_NO_2_ | 130.09 | HMDB0005960 | 0.97 | 1.04 | ↓*** | ↑^##^ |
| pos | D-Ornithine | C_5_H_12_N_2_O_2_ | 133.10 | HMDB0003374 | 0.90 | 1.12 | ↓** | ↑^###^ |

Note: the FC_M_ and FC_H_ values represent the fold change of the Model group compared to the Control group or H_AGE group, respectively. The levels of potential biomarkers were labeled with (↑) up-regulated and (↓) down-regulated. **p* < 0.05, ***p* < 0.01, ****p* < 0.001 compared with Control group; ^#^*p* < 0.05, ^##^*p* < 0.01, ^###^*p* < 0.001 compared with H_AGE group.
